# Experimental treatments to attenuate blood spinal cord barrier rupture in rats with traumatic spinal cord injury: A meta-analysis and systematic review

**DOI:** 10.3389/fphar.2022.950368

**Published:** 2022-08-23

**Authors:** Li Deng, Jun Qiao Lv, Lin Sun

**Affiliations:** Third Hospital of Shanxi Medical University, Shanxi Bethune Hospital, Shanxi Academy of Medical Sciences, Tongji Shanxi Hospital, Taiyuan, China

**Keywords:** spinal cord injury, blood spinal cord barrier, functional recovery, rat, meta-analysis

## Abstract

**Background:** Traumatic spinal cord injury (t-SCI) is a severe injury that has a devastating impact on neurological function. Blood spinal cord barrier (BSCB) destruction following SCI aggravates the primary injury, resulting in a secondary injury. A series of experimental treatments have been proven to alleviate BSCB destruction after t-SCI.

**Methods:** From a screen of 1,189 papers, which were retrieved from Pubmed, Embase, and Web of science, we identified 28 papers which adhered to strict inclusion and exclusion criteria. Evans blue (EB) leakage on the first day post-SCI was selected as the primary result. Secondary outcomes included the expression of tight junction (TJ) proteins and adhesion junction (AJ) proteins in protein immunoblotting. In addition, we measured functional recovery using the Basso, Beattie, Besnahan (BBB) score and we analyzed the relevant mechanisms to explore the similarities between different studies.

**Result:** The forest plot of Evans blue leakage (EB leakage) reduction rate: the pooled effect size of the 28 studies was 0.54, 95% CI: 0.47–0.61, *p* < 0.01. This indicates that measures to mitigate BSCB damage significantly improved in reducing overall EB leakage. In addition TJ proteins (Occludin, Claudin-5, and ZO-1), AJ proteins (P120 and β-catenin) were significantly upregulated after treatment in all publications. Moreover, BBB scores were significantly improved. Comprehensive studies have shown that in t-SCI, inhibition of matrix metalloproteinases (MMPs) is the most commonly used mechanism to mitigate BSCB damage, followed by endoplasmic reticulum (ER) stress and the Akt pathway. In addition, we found that bone marrow mesenchymal stem cell-derived exosomes (BMSC-Exos), which inhibit the TIMP2/MMP signaling pathway, may be the most effective way to alleviate BSCB injury.

**Conclusion:** This study systematically analyzes the experimental treatments and their mechanisms for reducing BSCB injury in the early stage of t-SCI. BMSC-Exos, which inhibit MMP expression, are currently the most effective therapeutic modality for alleviating BSCB damage. In addition, the regulation of MMPs in particular as well as the Akt pathway and the ER stress pathway play important roles in alleviating BSCB injury.

**Systematic Review Registration:** https://www.crd.york.ac.uk/prospero/display_record.php?ID=CRD42022324794.

## 1 Introduction

Traumatic spinal cord injury (t-SCI) is a devastating injury resulting in severe and permanent sensory and motor deficits (Karsy and Hawryluk, 2019). Following the initial primary injury to the spinal cord, a series of inflammatory and immune responses trigger further cellular damage and cascades known as secondary injuries (Gerzanich et al., 2009; Fan et al., 2018). Moreover, blood spinal cord barrier (BSCB) plays a key role in primary and secondary injury following SCI (Xin et al., 2021). After SCI, BSCB can restrict the entry of various harmful factors such as external inflammatory cells into the central nervous system, maintain local homeostasis, and play a considerable role in neuroprotection after SCI (Kaneko et al., 2006). Barrier function depends on vascular endothelial cells (ECs) and their additional components, such as pericytes and astrocyte, as well as junctions between endothelial cells, including tight junctions (TJ), adhesion junctions (AJ) and gap junctions (Stamatovic et al., 2016). These structures ensure the formation of a stable internal environment in the nervous system (Obermeier et al., 2013). Primary injury leads to the destruction of the local BSCB, infiltration of inflammatory cells, and migration to adjacent sites, followed by secretion of various matrix metallopeptidases (MMPs), cellular inflammatory factors, and reactive oxygen species (ROS’) leading to secondary damage and further destruction of the BSCB (Dumont et al., 2001; Alizadeh et al., 2019). Recently, multiple studies have explored a variety of methods to alleviate BSCB damage. The most common methods are inhibition of endoplasmic reticulum (ER) stress, inhibition of MMPs, promotion of haem oxygenase-1 (HO-1) activity, local cell transplantation, nanomedicine, hydrogels, and inhibition of the Akt pathway (Lee et al., 2015; Wang et al., 2017; Zhao et al., 2022).

**FIGURE 1:**
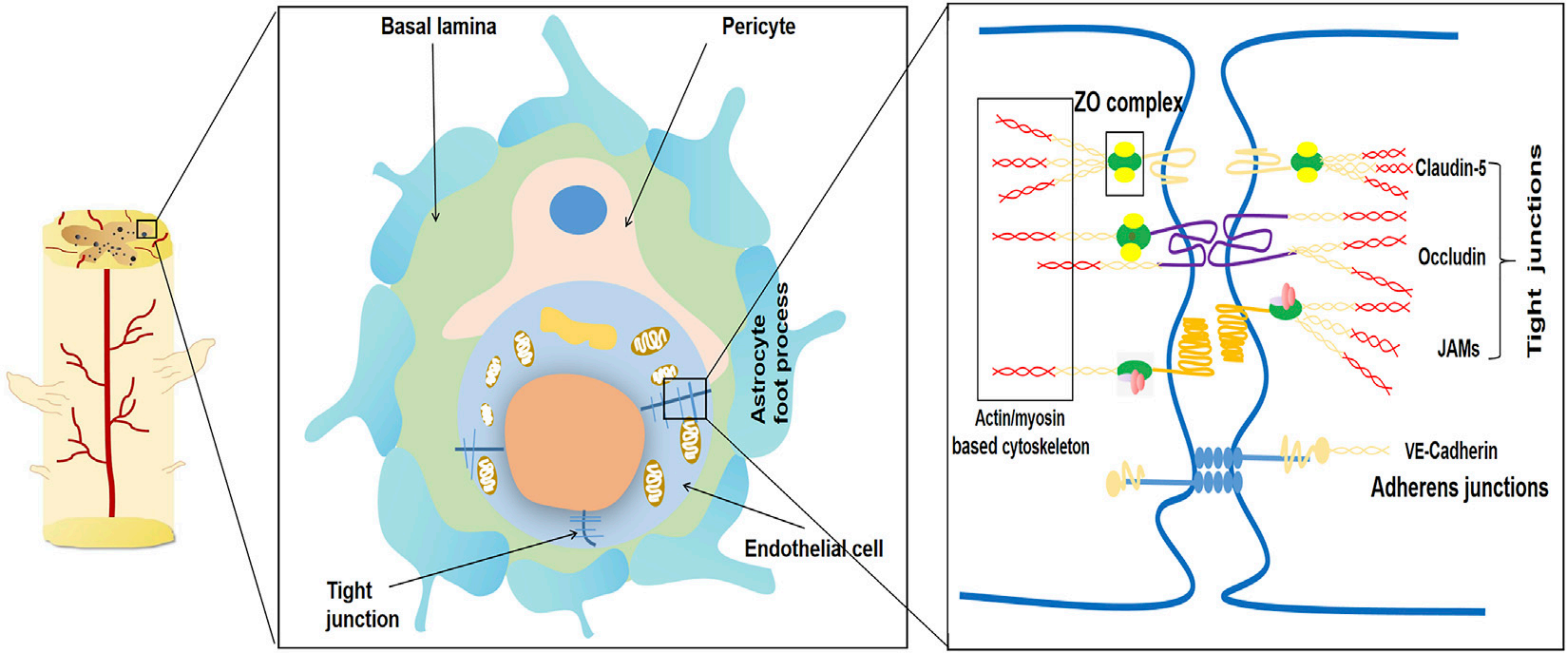
Schematic diagram of the main components of the blood-spinal cord barrier (BSCB). The blood-spinal cord barrier typically consists of endothelial cells with no pores attached to tight junctions (TJ), basal layer, pericytes, and astrocyte foot processes. The permeability of BSCB is mainly regulated by connexins and their complex molecular structure between endothelial cells. The typical tight junctions consist of Occludin, Claudins, ZO complex and adhesion junction. Adhesion junctions act as continuous bands in the plasma membrane and allow high levels of adhesion between endothelial cells.

For evaluating the integrity of BSCB, Evans blue (EB) is currently one of the most commonly used markers of BSCB integrity. EB is rapidly and completely bound to plasma albumin and its expression can be used as a measure of BSCB permeability (Nagaraja et al., 2008; Saunders et al., 2015). AJ is formed by the transmembrane adhesion proteins of the calmodulin family, which are widely dispersed in the vasculature. VE-cadherin is prevalent in endothelial cells where it binds β-catenin through its carboxy-terminal region, which can further stabilize AJ anchoring to actin (Vleminckx and Kemler, 1999). Another VE-cadherin chaperone, p120, which has various functions in different cells (e.g., increasing intercellular adhesion (Yap et al., 1998)). A series of TJ proteins, such as Occludin, Claudins, and zonula occludens protein 1 (ZO-1), together constitute the TJ molecule, which is closely implicated in the function of the BSCB. Occludin or Claudins bind to each other across the intercellular space and are further linked to the scaffold protein ZO-1. ZO-1 is one of the most important members of the membrane-associated guanylate kinase homologs, which can connect the cytoskeleton with TJ and AJ proteins. ZO proteins are linked to the actin/myosin cytoskeleton system through cingulin dimers within the cells. These TJ properties contribute to the diffusion barrier of the BSCB (Bazzoni and Dejana, 2004; Abbott et al., 2010) (Figure 1). TJ and AJ protein expression can affect the structural integrity and function of BSCB. Different scoring scales can be used to evaluate animal neurological function, with the most common one being the Basso, Beattie, Besnahan (BBB) locomotor score. The BBB score is a behavioral test developed in rats with an overall score of 21. This test is used to measure the functional recovery of hindlimbs in rats with spinal cord injury. In this test, following an operation, rats are allowed to walk in the field for 30 min every day. Healthy animals typically have a motor capacity score of 20–21, while animals with complete hindlimb paralysis are given a score of 0 (Basso et al., 1995).

MMPs belong to a family of zinc-dependent endonuclease proteases, which play a variety of roles in tissue remodeling and degradation. MMPs promote cell proliferation and migration, and play a role in angiogenesis, apoptosis, tumor invasion, fibrosis, and tissue repair (Wang and Khalil, 2018; Mahalanobish et al., 2020; Abdel-Hamid and Abass, 2021). However, overactivation of MMPs after SCI is detrimental and destroys the BSCB, leading to hematopoietic infiltration and apoptosis, and subsequent severe neurological dysfunction after SCI (Lee et al., 2018). Endogenous tissue inhibitors of MMPs (TIMPs) are key players in the regulation of MMP activity. TIMPs regulate the biological activity of MMPs without affecting their expression (Wright and Harding, 2009). Bone marrow mesenchymal stem cell transplantation has been shown to play a role in the recovery of neurological function after cerebral ischemia (Zhang et al., 2019). Exosomes are extracellular vesicles that are present in almost all cells. Bone marrow mesenchymal stem cell exosomes (BMSC-Exos) are easy to isolate and store, and have relatively stable biological properties (Rezaie et al., 2018). BMSC-Exos have been shown to attenuate cerebral ischemia-reperfusion injury-induced neuroinflammation (Liu et al., 2021; Zhou et al., 2022) and inhibition of pericyte pyrophosphorylation to protect the injured spinal cord (Zhou et al., 2022). Due to the remarkable therapeutic effect of BMSC-Exos, research on its treatment of spinal cord injury has received increasing attention in recent years.

The endoplasmic reticulum (ER) is a subcellular organelle, which stores intracellular Ca^2+^ (Kim et al., 2008). ER stress leads to inhibition of protein synthesis and depletion of ER Ca^2+^, ultimately increasing CHOP expression and activating caspase-dependent apoptosis (Lee et al., 2014a). In recent years, many studies have shown that inhibition of ER stress can upregulate TJ and AJ protein expression in a rat model of SCI, thereby improving BSCB integrity (Ye et al., 2016; He et al., 2017; Wang et al., 2018). The PI3K/Akt pathway is a cell survival transduction pathway, which is mediated by nerve cells (Zhang et al., 2015). This pathway is necessary for the stabilization of endothelial barrier function. Further, epidermal growth factor alleviates BSCB damage after spinal cord injury through the PI3K/Akt pathway (Zheng et al., 2016). Insulin also stabilizes endothelial barrier function through the PI3K/Akt pathway (Gunduz et al., 2010).

There is an urgent need to identify novel therapeutic strategies to alleviate BSCB damage following t-SCI. Various studies have demonstrated the utility of different treatments in alleviating BSCB breakdown, but there have been no systematic reviews of these treatments. Herein, we sought to systematically analyze the effective experimental treatment methods for BSCB interruption with the aim of identifying the most effective treatment methods and related mechanisms. Moreover, the data summarized here may be useful in future drug development and clinical translation studies.

## 2 Materials and methods

### 2.1 Inclusion and exclusion criteria

The included studies meet the following criteria: 1) studies published in English; 2) published in the last 10 years; 3) published in a peer reviewed journal; 4) studies conducted in rats. We had the following exclusion criteria: 1) studies that did not mention the primary endpoint (i.e., leakage of EB on day 1); 2) non-rat studies and *in vitro* studies; 3) review and systematic review articles; 4) lack of available full text publications. We first excluded literature based on the title and abstract and once shortlisted, two independent reviewers (Deng and Lv) performed full-text readings using an unblinded standard method for final selection. To limit the scope of search results, searches were limited to English-language literature published in the last 10 years because the majority of SCI research has been conducted in the last 10 years. Primary indicators included first-day EB leakage, because early EB leakage due to BSCB disruption was most pronounced on the first day (Lee et al., 2012; Fan et al., 2013; Du et al., 2016).

### 2.2 Search strategy

We searched three online databases (PubMed, Web of Science and Embase) for English studies published between 1st January 2012 and 30th June 2022. We used the Boolean operators to develop the following search strategy {[(spinal cord injury) AND (((BSCB) OR (blood–spinal cord barrier)) OR (BSCB))] AND (rat)} NOT (review), to retrieve relevant literature. After exporting to the literature management software, NoteExpress, duplicates were excluded and the literature was first initially screened against titles and abstracts based on inclusion and exclusion criteria. The shortlisted studies were again read in full by two separate reviewers. Subsequently, we generated the final included studies for downstream analysis.

### 2.3 Data extraction

Data were extracted from published literature using pre-designed tables to obtain basic research characteristics. This included author, date of publication, country in which the data were collected, strain of animal used, body weight of animal, segment of SCI, mode of SCI induction, mode of treatment, and main outcomes associated with BSCB. Secondly, we also reported amount of EB injected, the injection method, and the method of measuring the EB leakage.

All studies included EB content in sham, control, and treatment groups. In addition, 22 studies included control and treatment Occludin expression levels, 12 studies included ZO-1 levels, and eight studies included Claudin-5 levels. Moreover, seven and eight studies mentioned expression of cohesive p120 and β-catenin, respectively. However, in most studies, the relevant indicators were presented as graphs instead of raw data. Although some studies provided raw data, they could not be directly used for comparative analyses. This meant that we needed precise estimates of the actual numbers for these studies. Therefore, we extracted data from graphs provided in the literature using GetData software, which we then analyzed for accuracy. The relative percentage reduction in EB leakage was then calculated for each treatment group. From this, we summarized the relative expression changes of in AJ and TJ proteins. We also summarized other data from the included studies.

### 2.4 Risk of bias

Selected publications were assessed for risk of bias using the Systematic Review Centre for Laboratory animal Experimentation (SYRCLE)’s risk of bias tool (Hooijmans et al., 2014).

### 2.5 Meta-analysis

The main aim of the meta-analysis was to combine the effect sizes of the corresponding included studies to obtain a more precise estimate of the treatment effect size as well as a more direct observation of the magnitude of the effect sizes of the different treatment measures. We performed a meta-analysis of EB leakage rate, TJ protein- (Occludin, ZO-1, and Claudin-5), and AJ protein (β-catenin and p120) expression to comprehensively assess BSCB.

### 2.6 Statistical analysis

Heterogeneity between studies was determined using the inconsistency index I^2^. Given the heterogeneity and variability between studies, a random effects model was used. The effect sizes were represented by forest plots showing the effect sizes and their 95% confidence intervals for this study. This was performed using stata14 software, using a random effects model.

## 3 Results

### 3.1 Study selection

**FIGURE 2:**
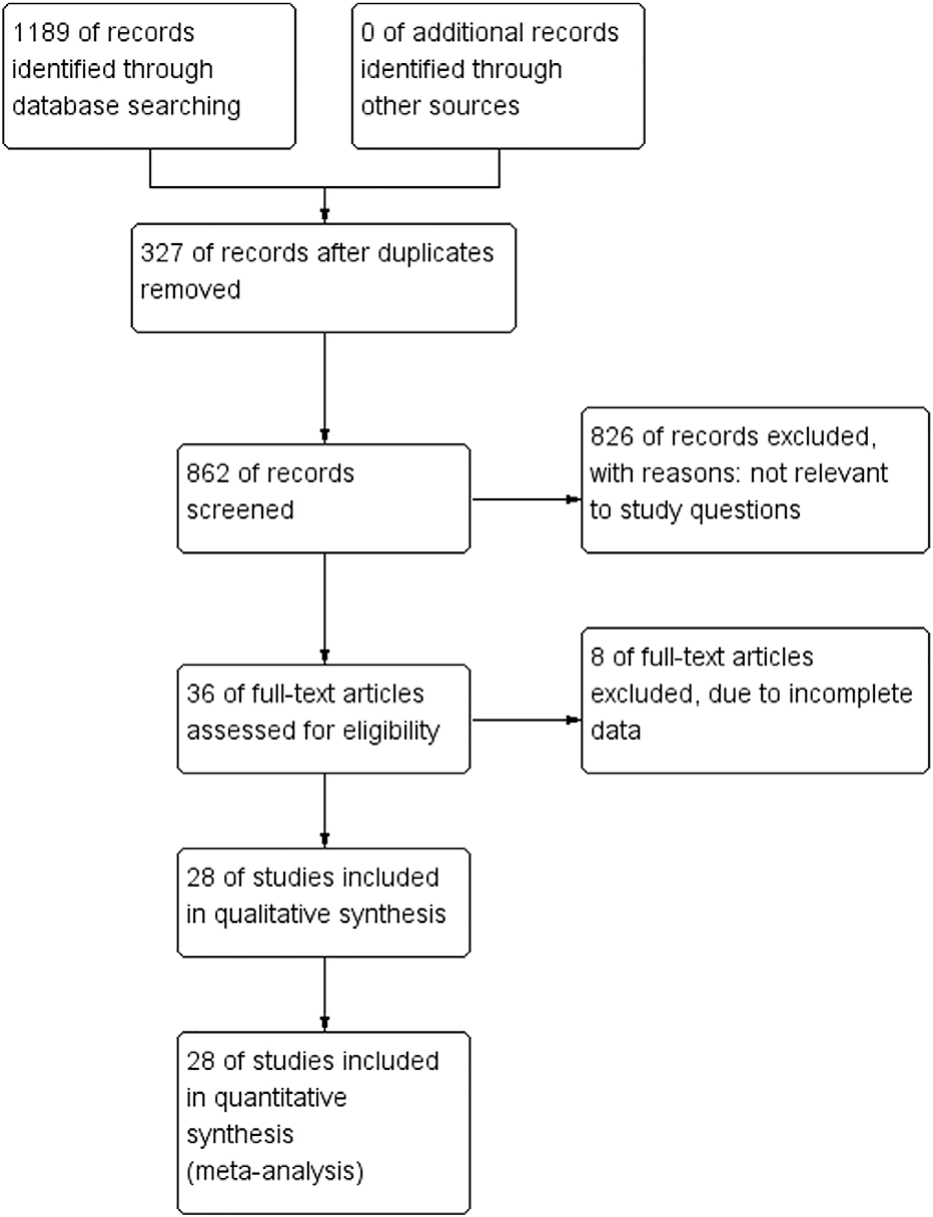
The PRISMA flow chart demonstrates the selection process for studies included in systematic reviews.

A systematic search yielded 1,189 results from Pubmed, Embase, and Web of science. Following deduplication, 862 articles remained for preliminary screening of titles and abstracts. Following our established inclusion and exclusion criteria, 826 studies were excluded for not meeting the inclusion exclusion criteria, leaving 36 for full text reading. After full-text screening, eight studies were excluded due to incomplete data. Therefore, 28 studies were included in this study. The inclusion exclusion process of the literature is represented by the PRISMA diagram (Figure 2).

### 3.2 Study characteristics

Table 1 shows study characteristics, summarizing the general characteristics of the studies included. Most publications were from China (53%), while the rest were conducted in Korea and Canada. Except for one study (Chio et al., 2021), all studies used Sprague Dawley rats as experimental animal models, although some rats were of different sexes. SCI was induced between T8 and T10 in every study except one (Chio et al., 2021). Although SCI was induced in all studies, the method used varied across different studies; 50% of studies used contusion, while the other 50% used compression. Different treatment regimens were used in each study but many treatment regimens acted on the same target (Table 1).

**TABLE 1:** Characteristics 506 of the included studies.

Study	Study site	Strain	Weight(g)	Level of SCI	Type of SCI	Treatment	Treatment type	Drug dosage	Way of administration	Treatment time	Main outcome measures
Chang et al. (2018)	China	female Sprague Dawley	280–320	T10	contusion	Ad-GFP-HO-1CΔ23	Enhance Ho-1 activity	7.9 × 108 PFUs	IT	7 days before SCI	EB, Occludin, Claudin5, BBB, HO-1
Chio et al. (2021)	Canada	female Wistar	unknown	C7-T1	Compression	hIgG	systemic immunosuppression	2 g/kg	iv	15 min, 1 h or 4 h after SCI	EB, ZO-1, Occludin, MMP-9, MPO, BBB
Gao et al. (2018)	China	male Sprague Dawley	250–300	T10	Compression	MANF	AKT Pathway	5 ug	ICV and IT	ICV 1 h after SCI and IT 48 h before SCI	EB, water content, BBB
He et al. (2017)	China	female Sprague Dawley	220–250	T9	Compression	LiCl	Inhibit ER stress	20 mg/kg	ip	2 h before SCI and daily post SCI	EB, Occludin, Claudin-5, P-120, β-catenin, ER stress, BBB
Huang et al. (2021)	China	female Sprague Dawley	200–220	T9	contusion	PA	AKT pathway and Inhibit ER Stress	10 mg/kg	ip	consecutive 56 days after SCI	EB, Claudin-5, β-catenin, ER stress, BBB
Joshi et al. (2020)	Korea	female Sprague Dawley	200–250	T10	Compression	CORM-2-SLN	AKT Pathway	10 mg/kg	ip	consecutive 8 days after SCI	EB, ZO-1, ZO-2, Occluding, Claudin-1, BBB
Kumar et al. (2018)	Korea	female Sprague Dawley	200–220	T10	Compression	MMP-8I	Reduce MMP-8	5 mg/kg	unknown	30 min after SCI	EB, Occludin, ZO-1, MMP-8, HO-1
Lee et al. (2012)	Korea	male Sprague Dawley	250–300	T9–T10	contusion	VPA	inhibit MMP-9	300 mg/kg	sc	twice a day for 5 days after SCI	EB, ZO-1, Occludin, MMP-2, MMP-9, MPO, ED-1, BBB
Lee et al. (2014a)	Korea	male Sprague Dawley	250–300	T9-T10	contusion	Ghrelin	inhibit MMP-9	80 μg/kg	ip	q6h for 1 day after SCI	EB, ZO-1, Occludin, MMP-2, MMP-9
Lee et al. (2015)	Korea	male Sprague Dawley	250–300	T9-T10	contusion	E2	inhibit MMP-9 and Inhibit ER stress	300 g/kg	iv	0 h,6 h, 24 h after SCI	Eb, ZO-1, Occludin, MMP-9, MMP-2, BBB, MPO, ED-1
Lee et al. (2016a)	Korea	male Sprague Dawley	250–270	T9-T10	contusion	Jmjd3 siRNA	inhibit MMP-3, and MMP-9	2 ul/site	IT	30 min after SCI	EB, MMP-2, MMP-3, MMP-9, Occludin, ZO-1, MPO, ED-1, BBB
Lee et al. (2016b)	Korea	male Sprague Dawley	250–300	T9-T10	contusion	fluoxetine and vitamin C	inhibit MMP-9	fluoxetine 1 mg/kg vitamin C 100 mg/kg	ip	immediately and then once a day for 14 d	EB, ZO-1, Occludin, MMP-2, MMP-9, MPO, ED-1, BBB
Lee et al. (2017)	Korea	male Sprague Dawley	250–300	T10	contusion	WIB-801C	inhibit MMP-9	50 mg/kg	po	2 and 8 h after SCI and then once a day for 2 weeks	EB, MMP-2, MMP-9, MPO, ED-1, BBB
Lee et al. (2018)	Korea	male Sprague Dawley	250–300	T9	Compression	MA	inhibit SUR1/TRPM4 and MMP-9	150 ug/kg	ip	immediately after SCI and then once a day for 5 d	EB, Occludin, ZO-1, MMP-2, MMP-9, MPO, ED-1, BBB
Park et al. (2019)	Korea	male Sprague Dawley	250–300	T9	contusion	PCA	inhibit SUR1/TRPM4 and MMP-9	50 mg/kg	ip	6 and 12 h after SCI and then once a day for 7 days	EB, Claudin-5, Occludin, ZO-1, MMP-2, MMP-9, MPO, ED-1, BBB
Park et al. (2020)	Korea	male Sprague Dawley	250–300	T9-T10	contusion	GA	inhibit Jmjd3 and MMP-9	50 mg/kg	ip	6 and 12 h after SCI and then once a day for 7 days	EB, ZO-1, Occludin, MMP-2, MMP-9, MPO, ED-1, BBB
Tong et al. (2018)	China	female Sprague Dawley	220–250	T9	Compression	LiCl	stimulating autophagic	20 mg/kg	ip	immediately after SCI	EB, Claudin-5, Occludin, BBB
Wang et al. (2017)	China	female Sprague Dawley	220–250	T9	contusion	aFGF-HP hydrogels	Inhibit the ER Stress	20 μg	oi	immediately after SCI	EB, P120, Occludin, ER stress, BBB
Wang et al. (2018)	China	female Sprague Dawley	200–220	T9	Compression	NaHS	Inhibit the ER Stress	5.6 mg/kg	ip	30 min before SCI and daily post SCI	EB, ER stress, Occludin, BBB, P120, β-catenin
Wang et al. (2020)	China	male Sprague Dawley	180–220	T10	contusion	VA-CN	Not mentioned	15 mg/kg	iv	once a day for 5 days after SCI	EB, BBB, TNF-a, IL-1b, Claudin-5
Xin et al. (2021)	China	female Sprague Dawley	220–250	T9	contusion	BMSC-Exos	inhibit TIMP2/MMP	100 μg	sc	once a day for 7 days after SCI	EB, TIMP2/MMP-2, MMP-9, Claudin-5, Occludin, ZO-1
Zheng et al. (2016)	China	female Sprague Dawley	220–250	T9	contusion	EGF	Akt pathway	60 ug/kg	sc	once a day for 7 days after SCI	EB, Claudin-5, Occludin, P120, β-catenin, BBB
Zheng et al. (2017)	China	female Sprague Dawley	220–250	T9	Compression	NBP	Inhibit the ER Stress	80 mg/kg	ig	once a day after SCI	EB, Claudin-5, Occludin, ER stress, BBB, P120, β-catenin
Zhou et al. (2016a)	China	female Sprague Dawley	220–250	T9	Compression	RA	activation of autophagic and inhibition of ER stress	15 mg/kg	ip	once a day for 14 days after SCI	EB, Claudin-5, Occludin, P120, β-catenin, ER stress, BBB
Zhou et al. (2016b)	China	female Sprague Dawley	220–250	T9	Compression	PBA	inhibit ER Stress	100 mg/kg	ip	once a day for 14 days after SCI	EB, Claudin-5, Occludin, P120, β-catenin, ER stress, BBB
Zhou et al. (2019)	China	male Sprague Dawley	250–300	T8-T10	Compression	BBG	Not mentioned	50 mg/kg	ip	twice a day for 7 days after SCI	EB, BBB
Feng et al. (2021)	China	female Sprague Dawley	250–300	T9	Compression	inhibit NETs	inhibit TRPV4	Clamidine: 50 mg/kg DNase1 5 mg/kg	Clamidine: ip DNase I: iv	1 h after SCI	EB, water content, BBB, Occludin, ZO-1
Park et al. (2022)	Korea	male Sprague Dawley	250–300	T9-T10	Compression	CAR	inhibit TRPM7	50 mg/kg	ip	0 and 12 h after SCI, and then once a day for 7 days	EB, Occludin, ZO-1, TRPM7, BBB, MPO, ED-1

**Ad-GFP-HO-1CΔ23:** adenoviral haem oxygenase-1, fragments lacking 23 amino acids at the C-terminus **higG:** human immunoglobulin G **MANF:** Mesencephalicastrocyte-derived neurotrophic factor **LiCl:** Lithium chloride **PA:** patchouli alcohol **CORM-2-SLN:** Carbon monoxide-releasing molecule-2, incorporated solid lipid nanoparticle. **MMP-8I:** Metalloproteinase-8, Inhibition **VPA:** valproic acid **E2:**17β-Estradiol **MA:** mithramycin A **PCA:** protocatechuic acid **GA:** Gallic acid **aFGF-HP, hydrogels:** Acidic fifibroblast growth-loaded thermosensitive heparin_x0002_poloxamer hydrogel **VA-CN:** valproic acid labeled chitosan nanoparticles **BMSC-Exos:** bone marrow mesenchymal stem cell derived exosomes **EGF:** epidermal growth factor **NBP:** Dl3-n-butylphthalide **RA:** Retinoicacid **PBA:** Phenylbutyrate **BBG:** Brilliant blue G **NETs:** neutrophils produce neutrophil extracellular traps **CAR:** carvacrol **iv:** intravenous injected **ip:** intraperitoneal injected **IT:** intrathecally injected **sc:** cinjected subcutaneously **oi: o**rthotopically injected **Ig:** coral gavage **ICV:** intracerebroventricular injections.

### 3.3 Literature quality assessment

Risk of bias analysis was performed on the information provided from the 28 included studies using SYRCLE’s risk of bias tool. The scale covers 10 domains, which, according to the information provided in the publications, were classified as “Yes”, “Unclear”, or “No” (Table 2). With the exception of one study (Chio et al., 2021), no other studies clearly stated their specific method of randomization. None of the publications mentioned allocation concealment and the implementation of blinding. Almost all publications described the two groups of animals as being similar in baseline characteristics (e.g., sex and body weight), with the exception of one study (Chio et al., 2021). In addition, 39% of the publications described the two groups of animals as being housed in the same conditions. No the studies clearly stated whether animals were selected at random for outcome assessment or selectively reported their findings. Overall, bias in all studies except for Chio et al. (2021), was considered moderate to high risk, which may have affected the quality of these studies.

**TABLE 2:** Risk of bias in studies.

Study	1	2	3	4	5	6	7	8	9	10
Chang et al. (2018)	Unclear	Yes	Unclear	Unclear	Unclear	Unclear	Unclear	Unclear	Yes	Unclear
Chio et al. (2021)	Yes	Unclear	Yes	Yes	Yes	Unclear	Yes	Yes	Yes	Unclear
Gao et al. (2018)	Unclear	Yes	Unclear	Unclear	Unclear	Unclear	Unclear	Unclear	Yes	Unclear
He et al. (2017)	Unclear	Yes	Unclear	Unclear	Unclear	Unclear	Unclear	Unclear	Yes	Unclear
Huang et al. (2021)	Unclear	Yes	Unclear	Unclear	Unclear	Unclear	Unclear	Unclear	Yes	Unclear
Joshi et al. (2020)	Unclear	Yes	Unclear	Yes	Unclear	Unclear	Unclear	Unclear	Yes	Unclear
Kumar et al. (2018)	Unclear	Yes	Unclear	Yes	Unclear	Unclear	Unclear	Unclear	Yes	Unclear
Lee et al. (2012)	Unclear	Yes	Unclear	Unclear	Unclear	Unclear	Unclear	Unclear	Yes	Unclear
Lee et al. (2014b)	Unclear	Yes	Unclear	Unclear	Unclear	Unclear	Unclear	Unclear	Yes	Unclear
Lee et al. (2015)	Unclear	Yes	Unclear	Unclear	Unclear	Unclear	Unclear	Unclear	Yes	Unclear
Lee et al. (2016a)	Unclear	Yes	Unclear	Unclear	Unclear	Unclear	Unclear	Unclear	Yes	Unclear
Lee et al. (2016b)	Unclear	Yes	Unclear	Unclear	Unclear	Unclear	Unclear	Unclear	Yes	Unclear
Lee et al. (2017)	Unclear	Yes	Unclear	Yes	Unclear	Unclear	Unclear	Unclear	Yes	Unclear
Lee et al. (2018)	Unclear	Yes	Unclear	Unclear	Unclear	Unclear	Unclear	Unclear	Yes	Unclear
Park et al. (2019)	Unclear	Yes	Unclear	Yes	Unclear	Unclear	Unclear	Unclear	Yes	Unclear
Park et al. (2020)	Unclear	Yes	Unclear	Unclear	Unclear	Unclear	Unclear	Unclear	Yes	Unclear
Tong et al. (2018)	Unclear	Yes	Unclear	Unclear	Unclear	Unclear	Unclear	Unclear	Yes	Unclear
Wang et al. (2017)	Unclear	Yes	Unclear	Unclear	Unclear	Unclear	Unclear	Unclear	Yes	Unclear
Wang et al. (2018)	Unclear	Yes	Unclear	Yes	Unclear	Unclear	Unclear	Unclear	Yes	Unclear
Wang et al. (2020)	Unclear	Yes	Unclear	Unclear	Unclear	Unclear	Unclear	Unclear	Yes	Unclear
Xin et al. (2021)	Unclear	Yes	Unclear	Yes	Unclear	Unclear	Unclear	Unclear	Yes	Unclear
Zheng et al. (2016)	Unclear	Yes	Unclear	Yes	Unclear	Unclear	Unclear	Unclear	Yes	Unclear
Zheng et al. (2017)	Unclear	Yes	Unclear	Unclear	Unclear	Unclear	Unclear	Unclear	Yes	Unclear
Zhou et al. (2016b)	Unclear	Yes	Unclear	Yes	Unclear	Unclear	Unclear	Unclear	Yes	Unclear
Zhou et al. (2016a)	Unclear	Yes	Unclear	Yes	Unclear	Unclear	Unclear	Unclear	Yes	Unclear
Zhou et al. (2019)	Unclear	Yes	Unclear	Unclear	Unclear	Unclear	Unclear	Unclear	Yes	Unclear
Feng et al. (2021)	Unclear	Yes	Unclear	Yes	Unclear	Unclear	Unclear	Unclear	Yes	Unclear
Park et al. (2022)	Unclear	Yes	Unclear	Unclear	Unclear	Unclear	Unclear	Unclear	Yes	Unclear

1. Sequence generation 2. Baseline characteristics 3. Allocation concealment 4. Random housing 5.Blinding (Performance bias) 6.Random outcome assessment 7.Blinding (Detection bias) 8. Incomplete outcome data 9. Selective outcome reporting 10. Other sources of bias.

### 3.4 Results of individual studies

We have summarized the individual results of each study to explore the outcomes associated with the different treatment options they chose and the similarities of the various treatment options (Table 1 and Table 3). In addition, 11 studies reported that inhibition of MMPs reduced BSCB damage compared to SCI and eight studies demonstrated that inhibition of ER stress could reduce BSCB leakage. Further, four studies also confirmed the effect of inhibiting the Akt pathway on BSCB (Table 1). Chang et al. (2018) demonstrated that GFP-HO-1CΔ23 enhances HO-1 activity to protect BSCB. Wang et al. (2017) also showed that A Thermosensitive Heparin-Poloxamer Hydrogel Bridges aFGF (aFGF-HP) hydrogels inhibit ER stress to alleviate BSCB rupture. Nine publications mentioned that reduction of BSCB damage also reduces neutrophil and macrophage infiltration into the BSCB in the blood, thereby reducing neuroinflammation. In addition, 21 studies have also tested neurological recovery in rats, using different methods, of which BBB assessment is the most commonly used method (Table 3). All studies showed significant improvement in BBB scores after treatment. Taken together, reducing BSCB disruption improved prognosis for the animals. Finally, we also investigated other less common results that were used to support respective research hypotheses.

**TABLE 3:** Results of individual studies.

Study	Summary of individual outcomes included in studies
Chang et al. (2018)	Increases the nuclear expression of HO-1
Chio et al. (2021)	Reduced spinal cord neutrophil infiltration at 24 h, Reduced the blood neutrophil population, Increase neutrophil population and pro-inflammatory chemokines/cytokines, Enhanced neurobehavioral recovery
Gao et al. (2018)	Akt/MDM-2/p53 pathway, Reduce apoptosis, Reduce spinal cord edema
He et al. (2017)	Improved locomotor function (BBB scale), Prevents endothelial cells damage, Inhibit ER stress
Huang et al. (2021)	Inhibit ER stress, Inhibits endothelial apoptosis, Reduce mitochondrial damage, Promote functional recovery, PI3K/AKT pathway
Joshi et al. (2020)	AKT pathway, Anti-inflammatory effects, Attenuate apoptotic, Protect endothelial cells, Prevents glial scar formation
Kumar et al. (2018)	Reduce inflammation, Reduce MMP-8
Lee et al. (2012)	Inhibit MMP-9, Reduce inflammation, Inhibit apoptotic, Improve functional recovery
Lee et al. (2014c)	Inhibit MMP-9 expression, Inhibits SUR1 and TrpM4 expression, Reduce hemorrhage, Inhibit the infiltration of neutrophils and macrophages
Lee et al. (2015)	Reduce hemorrhage, Inhibit MMP-9, Inhibits SUR1 and TrpM4 expression, Inhibits the expression of inflammatory mediators and chemokines, Inhibits the infiltration of neutrophils and macrophages, Inhibit ER stress
Lee et al. (2016a)	Relieve MMP-3 and MMP-9 expression, NF-κB, Inhibits the infiltration of blood cells and the expression of cytokines and inflammatory mediators, Inhibits neuronal apoptosis, Improves functional recovery, Reduce axon and myelin loss
Lee et al. (2016b)	Relieve MMP-9 expression, Inhibit the infiltration of neutrophils and macrophages, Inhibit apoptosis
Lee et al. (2017)	Inhibit MMP-9 expression, Inhibit blood cell infiltration, Reduce the expression of inflammatory mediators and chemokines, Inhibit apoptosis, Improves functional recovery, Reduce axon loss and lesion volume
Lee et al. (2018)	Inhibit cell death of neurons and oligodendrocytes inhibit hemorrhage, Inhibit MMP-9 expression, Inhibit SUR1 and TRPM4 expression, Inhibit the infiltration of neutrophils and macrophages, Inhibit inflammatory, Improve functional recovery, Attenuate loss of myelin and axon
Park et al. (2019)	Inhibit apoptosis, Inhibit MMP-9 expression, Inhibit SUR1 and TrpM4 expression, Inhibit the infiltration of neutrophils and macrophages, Inhibit inflammatory, Improve functional recovery, Alleviate axon and myelin loss
Park et al. (2020)	Inhibit the expression of Jmjd3, Inhibit Mmp-3 and MMP-9 expression, Inhibit the infiltration of neutrophils and macrophages, Inhibit inflammatory, Improve functional recovery, Alleviate axon and myelin loss
Tong et al. (2018)	Improve functional recovery, Promote autophagic flux
Wang et al. (2017)	Reduce the apoptosis, Promote remyelination and axonal rehabilitation, Attenuates the reactive astrogliosis, inhibit inflammatory, Promotes axonal generation across the glial scar, Improve functional recovery, Inhibit the ER Stress
Wang et al. (2018)	Induced ER Stress and autophagy
Wang et al. (2020)	Enhanced the function and tissue recovery, Reduced astrocytic reactivity, Promote neuroprotection, Inhibit inflammation
Xin et al. (2021)	TIMP2/MMP Pathway, Improve functional recovery
Zheng et al. (2016)	PI3K/Akt pathway
Zheng et al. (2017)	Accelerate locomotion recovery, Inhibit ER stress
Zhou et al. (2016b)	Improve functional recovery, Induce autophagic, Inhibit ER stress
Zhou et al. (2016a)	Inhibit ER Stress, Inhibit apoptosis in ECs
Zhou et al. (2019)	Enhanced the function and tissue recovery, Inhibit inflammation
Feng et al. (2021)	Relieve neuroinflammation and edema, Reduces cell death, Reduce scar formation, Enhanced the function and tissue recovery, Inhibit TRPV4 express
Park et al. (2022)	Inhibits BSCB disruption and preserves TJ integrity, Inhibits the infiltration of neutrophils and macrophages and the expression, Inhibits the apoptotic of neurons and oligodendrocytes, Increases functional recovery, Inhibits the loss of axon and myelin

### 3.5 Attenuation of blood spinal cord barrier breakdown

Reduction in EB leakage after treatment reported in included studies ranged from 32 to 90% (Table 4). This finding could be used to determine the effectiveness of each treatment option. Numerous studies have reported that EB leakage is most pronounced on the first day after SCI in rats (Fan et al., 2013; Lee et al., 2014c; Yu et al., 2014). In this study, EB leakage on the first day after injury was chosen as the criterion for assessing BSCB damage. The secondary indicators of BSCB analysis were significantly different between studies, but expression of AJ and TJ proteins was commonly used. Therefore, we divided the studies into three relevant indicators: primary indicator EB leakage and expression of secondary indicators of TJ and AJ proteins to investigate possible optimal treatment options (Tables 4–6). BMSC-Exos were the most effective method of alleviating EB leakage, and significantly inhibited destruction of BSCB by inhibiting the TIMP2/MMP pathway. Xin et al. (2021) reported that EB leakage was reduced by 90%, while expression of AJ and TJ proteins were significantly increased after BMSC-Exos treatment (Occludin: exp 1.28 ± 0.09 vs. sci 0.46 ± 0.03; ZO-1: exp 0.79 ± 0.07 vs. sci 0.16 ± 0.01; Claudin-5: exp 1.11 ± 0.06 vs. sci 0.26 ± 0.02; β-catenin exp 1.14 ± 0.07 vs. sci 0.14 ± 0.01) (Tables 4–6). Eight papers mentioned the effect of ER stress on BSCB, in which the reduction rate of EB leakage was between 39 and 67%, and expression of AJ and TJ proteins was also significantly upregulated (Table 1 and Tables 4–6). In addition, four studies demonstrated the effect of the Akt pathway on BSCB, mitigating EB leakage rate by 56–74% (Table 1 and Table 4).

**TABLE 4:** EB leakage mitigation.

Study	Treatment	Units of measurement	Weight (g)	EB concentration (%)	EB injection volume	EB injection method	*n*	EB leakage mitigation rate
Chang et al. (2018)	Ad-GFP-HO-1CΔ23	intensity	280–320	2	200 mg/kg	iv	6	0.76
Chio et al. (2021)	hIgG 15min after sci	ug/ml	Unknown	2	1 ml	iv	6	0.32
	hIgG 1 h after sci	ug/ml	Unknown	2	1 ml	iv	6	0.34
Gao et al. (2018)	MANF	ug/g	250–300	3	45 mg/kg	iv	6	0.56
He et al. (2017)	LiCl	ug/g	220–250	2	4 ml/kg	iv	4	0.45
Huang et al. (2021)	PA	ug/g	200–220	2	4 ml/kg	iv	8	0.52
Joshi et al. (2020)	CORM-2-SLN	intensity	200–250	2	0.5 ml	iv	3	0.74
Kumar et al. (2018)	MMP-8I	ug/g	200–220	2	0.5 ml	iv	3	0.35
Lee et al. (2012)	VPA	ug/g	250–300	2	5 ml	ip	5	0.49
Lee et al. (2014a)	Ghrelin	ug/g	250–300	2	0.5 ml	iv	5	0.55
Lee et al. (2015)	E2	ug/g	250–300	2	5 ml	ip	5	0.58
Lee et al. (2016a)	Jmjd3 siRNA	ug/g	250–270	2	5 ml	ip	5	0.39
Lee et al. (2016b)	fluoxetine and vitamin C	ug/g	250–300	2	5 ml	ip	5	0.49
Lee et al. (2017)	WIB-801C	ug/g	250–300	2	5 ml	ip	5	0.39
Lee et al. (2018)	MA	ug/g	250–300	2	5 ml	ip	5	0.49
Park et al. (2019)	PCA	ug/g	250–300	2	5 ml	ip	3	0.78
Park et al. (2020)	GA	ug/g	250–300	2	5 ml	ip	5	0.63
Tong et al. (2018)	LiCl	ug/g	220–250	2	1 ml	iv	4	0.33
Wang et al. (2017)	aFGF-HP hydrogels	ug/g	220–250	2	4 ml/kg	iv	4	0.66
Wang et al. (2018)	NaHS	intensity	200–220	2	2 ml/kg	iv	5	0.62
Wang et al. (2020)	VA-CN	ug/g	180–220	2	Unknown	iv	4	0.55
Xin et al. (2021)	BMSC-Exos	ug/g	220–250	2	2 ml/kg	iv	4	0.90
Zheng et al. (2016)	EGF	ug/g	220–250	2	2 ml/kg	iv	4	0.62
Zheng et al. (2017)	NBP	ug/g	220–250	2	2 ml/kg	iv	5	0.67
Zhou et al. (2016b)	RA	ug/g	220–250	2	1 ml	iv	5	0.39
Zhou et al. (2016a)	PBA	ug/g	220–250	2	1 ml	iv	4	0.45
Zhou et al. (2019)	BBG	ug/g	250–300	2	2 ml/kg	iv	10	0.42
Feng et al. (2021)	Cl-amidine	ug/g	250–300	2	5 ml/kg	iv	6	0.39
	DNase I	ug/g	250–300	2	5 ml/kg	iv	6	0.52
Park et al. (2022)	CAR	ug/g	250–300	2	5 ml	ip	5	0.80

iv: intravenous injected; ip: intraperitoneal injected; Notes of treatment are in Table 1.

**TABLE 5:** Expression of TJ

Study	Treatment	Sci		Exp	Sci		Exp	Sci		Exp
		**Occludin**			**ZO-1**			**Claudin5**		
Chang et al. (2018)	Ad-GFP-HO-1CΔ23	0.12 ± 0.01	VS.	0.98 ± 0.01				0.05 ± 0.01	VS.	1.02 ± 0.04
Chio et al. (2021)	hIgG 15min after sci	0.35 ± 0.06	VS.	0.88 ± 0.1	0	VS.	0.75 ± 0.1			
	hIgG 1 h after sci	0.42 ± 0.08	VS.	1.01 ± 0.08	0	VS.	0.67 ± 0.04			
	hIgG 4 h after sci	0.4 ± 0.08	VS.	0.91 ± 0.08	0	VS.	0.83 ± 0.09			
He et al. (2017)	LiCl	0.48 ± 0.09	VS.	0.83 ± 0.05				0.31 ± 0.03	VS.	0.61 ± 0.08
Huang et al. (2021)	PA							0.33 ± 0.05	VS.	0.91 ± 0.09
Joshi et al. (2020)	CORM-2-SLN	0.25 ± 0.09	VS.	1.12 ± 0.07	0.52 ± 0.06	VS.	0.99 ± 0.01			
Kumar et al. (2018)	MMP-8I	0.46 ± 0.04	VS.	0.65 ± 0.02	0.73 ± 0.02	VS.	0.87 ± 0.9			
Lee et al. (2014b)	VPA	0.21 ± 0.05	VS.	0.51 ± 0.07	0.17 ± 0.04	VS.	0.3 ± 0.05			
Lee et al. (2015)	Ghrelin	0.33 ± 0.04	VS.	0.58 ± 0.06	0.39 ± 0.06	VS.	0.65 ± 0.05			
Lee et al. (2016a)	E2	0.14 ± 0.06	VS.	0.63 ± 0.08	0.35 ± 0.06	VS.	0.62 ± 0.06			
Lee et al. (2016b)	Jmjd3 siRNA	0.31 ± 0.09	VS.	0.86 ± 0.1	0.6 ± 0.72	VS.	0.98 ± 0.13			
Lee et al. (2018)	fluoxetine and vitamin C	0.34 ± 0.06	VS.	0.79 ± 0.09	0.19 ± 0.1	VS.	0.51 ± 0.11			
Park et al. (2019)	PCA	0.3 ± 0.18	VS.	0.94 ± 0.13	0.49 ± 0.12	VS.	0.85 ± 0.12			
Park et al. (2020)	GA	0.53 ± 0.06	VS.	0.83 ± 0.03	0.6 ± 0.05	VS.	0.9 ± 0.06			
Tong et al. (2018)	LiCl	0.49 ± 0.08	VS.	0.82 ± 0.06						
Wang et al. (2017)	aFGF-HP hydrogels	0.39 ± 0.01	VS.	1 ± 0.04						
Wang et al. (2018)	NaHS	0.44 ± 0.05	VS.	0.72 ± 0.06						
Xin et al. (2021)	BMSC-Exos	0.46 ± 0.03	VS.	1.28 ± 0.09	0.16 ± 0.01	VS.	0.79 ± 0.07	0.26 ± 0.02	VS.	1.11 ± 0.06
Zheng et al. (2016)	EGF	0.57 ± 0.05	VS.	0.9 ± 0.07				0.68 ± 0.03	VS.	0.93 ± 0.02
Zheng et al. (2017)	NBP	0.22 ± 0.05	VS.	0.48 ± 0.05				0.5 ± 0.03	VS.	0.84 ± 0.05
Zhou et al. (2016b)	RA	0.55 ± 0.06	VS.	0.79 ± 0.11				0.5 ± 0.08	VS.	0.91 ± 0.08
Zhou et al. (2016a)	PBA	0.32 ± 0.06	VS.	0.68 ± 0.06				0.46 ± 0.04	VS.	0.72 ± 0.03
Feng et al. (2021)	Cl-amidine	0.32 ± 0.11	VS.	0.44 ± 0.07	0.35 ± 0.08	VS.	0.59 ± 0.18			
	DNase I	0.34 ± 0.09	VS.	0.58 ± 0.18	0.32 ± 0.12	VS.	0.68 ± 0.17			
Park et al. (2022)	CAR	0.62 ± 0.03	VS.	0.93 ± 0.07	0.67 ± 0.04	VS.	0.93 ± 0.05			

Treatment: Drugs used in the Exp group Sci: spinal cord injury group Exp: experimental group Occludin: The expression level of the Occludin proteins (% of sham) ZO-1 The expression level of the ZO-1 proteins (% of sham) Claudin5: The expression level of the Claudin5 proteins (% of sham).

**TABLE 6:** Expression of AJ.

Study	Treatment	Sci		Exp	Sci		Exp
		**β-catenin**			**P120**		
He et al. (2017)	LiCl	0.43 ± 0.05	VS.	0.81 ± 0.09	0.45 ± 0.05	VS.	0.78 ± 0.06
Huang et al. (2021)	PA	0.35 ± 0.12	VS.	0.9 ± 0.21			
Wang et al. (2017)	aFGF-HP hydrogels				0.33 ± 0.02	VS.	1.11 ± 0.06
Wang et al. (2018)	NaHS	0.49 ± 0.07	VS.	0.76 ± 0.05	0.39 ± 0.06	VS.	0.76 ± 0.06
Xin et al. (2021)	BMSC-Exos	0.14 ± 0.01	VS.	1.14 ± 0.07			
Zheng et al. (2016)	EGF	0.67 ± 0.07	VS.	1 ± 0.06	0.52 ± 0.06	VS.	0.83 ± 0.07
Zheng et al. (2017)	NBP	0.38 ± 0.06	VS.	0.65 ± 0.04	0.45 ± 0.06	VS.	0.85 ± 0.07
Zhou et al. (2016b)	RA	0.8 ± 0.04	VS.	0.91 ± 0.07	0.2 ± 0.08	VS.	0.75 ± 0.07
Zhou et al. (2016a)	PBA	0.51 ± 0.01	VS.	0.77 ± 0.04	0.4 ± 0.04	VS.	0.86 ± 0.06

Treatment: Drugs used in the Exp group Sci: spinal cord injury group Exp: experimental group β-catenin: The expression level of the β-catenin proteins (% of sham) P120 The expression level of the P120 proteins (% of sham).

### 3.6 Meta-analysis

#### 3.6.1 Evans blue leakage

**FIGURE 3:**
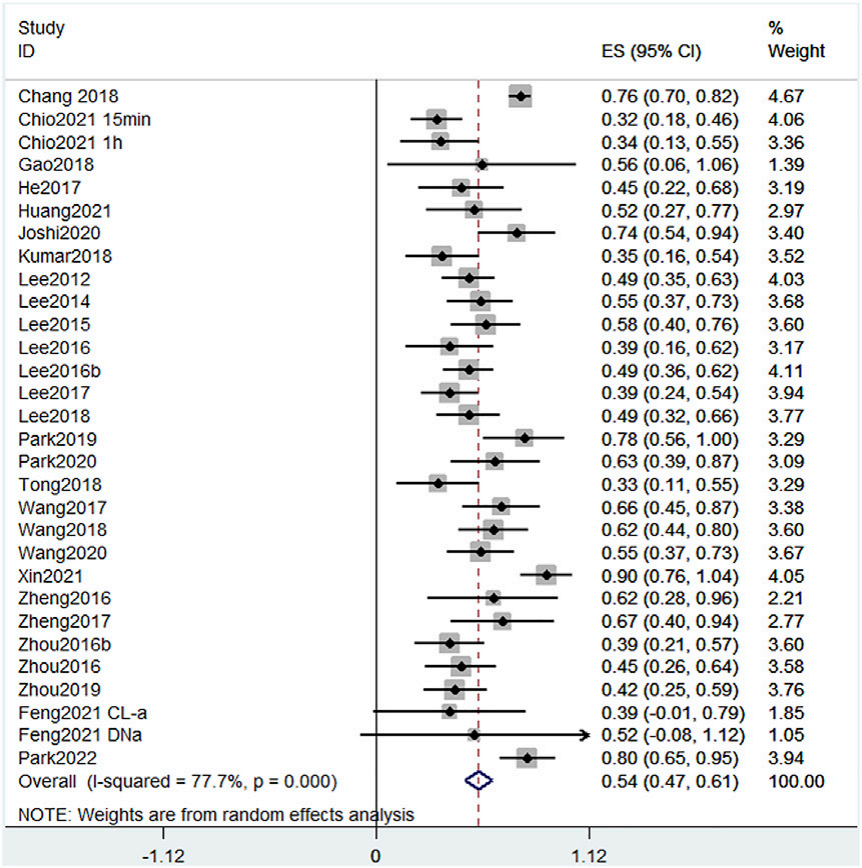
Effect size model for EB leakage at 24 h post-treatment. Black dots indicate weighted effect sizes for treatment regimens and error bars indicate 95% confidence intervals for each outcome. Heterogeneity of the study is indicated by the I2 statistic. *p* < 0.001 indicates statistical significance. At 24 h, studies presented a heterogeneity I2 score of 77.7%, also indicating a high level of heterogeneity. The effect size of each study except Feng et al. (2021) was also statistically positive, indicating that the treatment significantly reduced EB leakage in SCI.

Given that the injection method, injection volume, and rat body weight of EB in different publications were inconsistent, and the data in the literature are not available for direct comparison, we used the error propagation formula to calculate the mean and standard deviation of the EB leakage reduction rate, and then meta-analyzed the rate of reduction in EB leakage after each treatment. In random-effects meta-analyses, between-study heterogeneity was assessed by I^2^ values (Figure 3). In the leakage of EB, the heterogeneity among studies was 77.7% when meta-analyzed using the I^2^ statistic, indicating a high degree of heterogeneity between studies. We considered that the high heterogeneity is due to differences in the extent of injury, measurement methods, and baseline conditions in rats across studies. All treatments, except one (Feng et al., 2021), had statistically significant positive effects, indicating that the included treatment regimens had a positive effect on mitigating BSCB destruction. The forest plot showed that Xin et al. (2021) had the greatest reduction (90%) in EB leakage, treated with BMSC-Exos. Further, Joshi et al. (2020) treated with CORM-2-SLN (74%), Chang et al. (2018) treated with GFP-HO-1CΔ23 (76%), Park et al. (2022) treated with carvacrol (80%), and Park et al. (2019) treated with Protocatechuic acid (78%). These studies all achieved >70% reduction in EB leakage.

#### 3.6.2 Tight junction and adhesion junction protein expression

**FIGURE 4:**
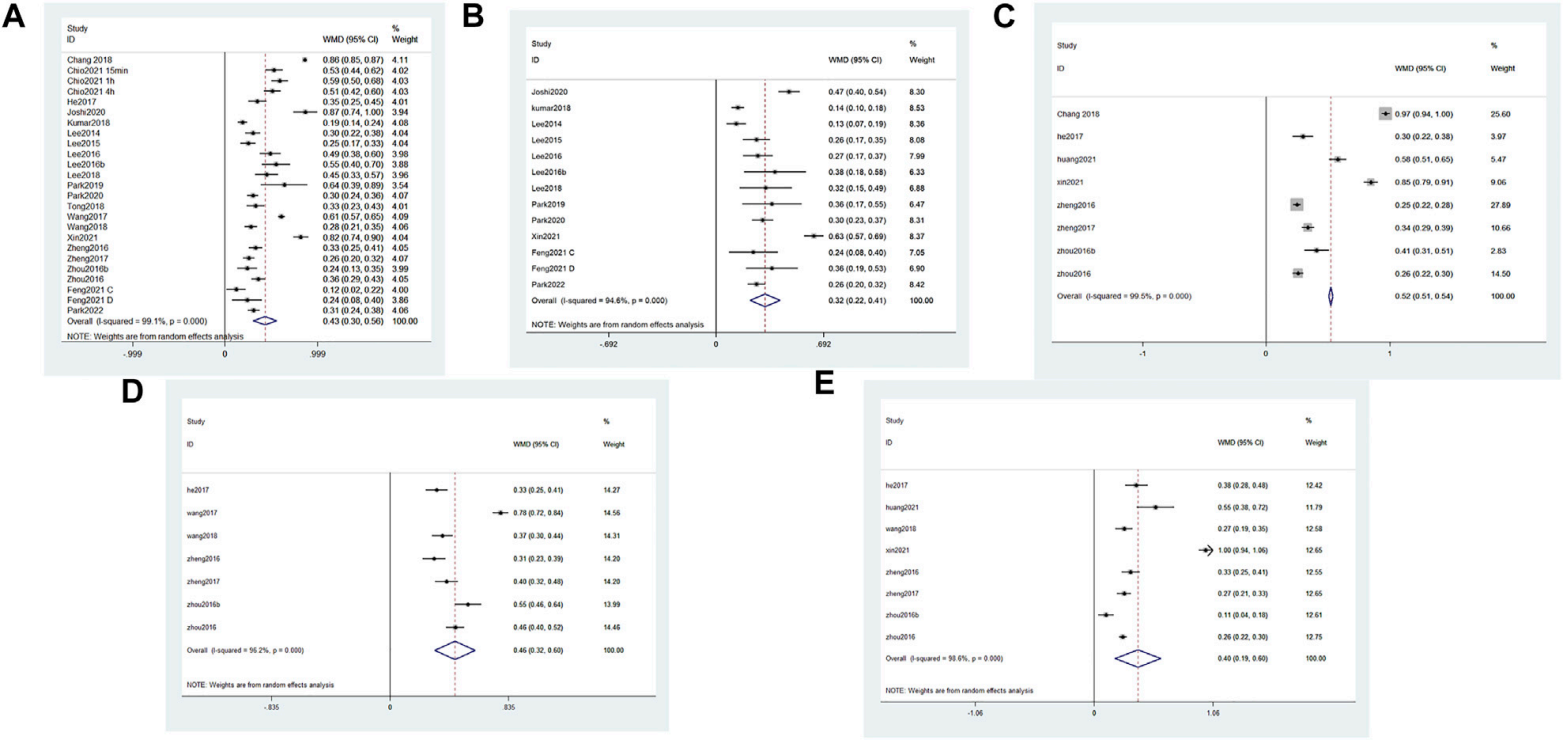
Forest plot of increased TJ and AJ protein expression in different studies. Black dots indicate weighted effect sizes of treatment regimens, and error bars indicate 95% confidence intervals for each outcome. The diamond box is the overall effect size and its confidence interval. **(A)** Forest plot of Occludin expression **(B)** Forest plot of ZO-1 expression **(C)** Forest plot of Claudin5 expression **(D)** Forest plot of P120 expression **(E)** Forest plot of β-catenin expression.

We summarized the changes in TJ and AJ protein expression as measured by western blot using forest plots to allow a more comprehensive assessment of BSCB. We found that all treatments significantly increased expression of TJ and AJ proteins in the experimental group (Figure 4). Occludin protein expression increased the most among the four treatments: CORM-2-SLN (0.87), GFP-HO-1CΔ23 (0.86), Protocatechuic acid (0.64), and BMSC-Exos (0.82). In two studies, the increase in ZO-1 was most pronounced. The authors used BMSC-Exos (0.63) and CORM-2-SLN (0.47), respectively. Claudin-5 expression increased the most after GFP-HO-1CΔ23 and BMSC-Exos treatment, reaching 0.97 and 0.85, respectively. In addition, aFGF-HP hydrogels treatment increased the most P120 expression up to 78%. β-Catenin increased up to 100% after treatment with BMSC-Exos (Figure 4).

#### 3.6.3 BBB score

**FIGURE 5:**
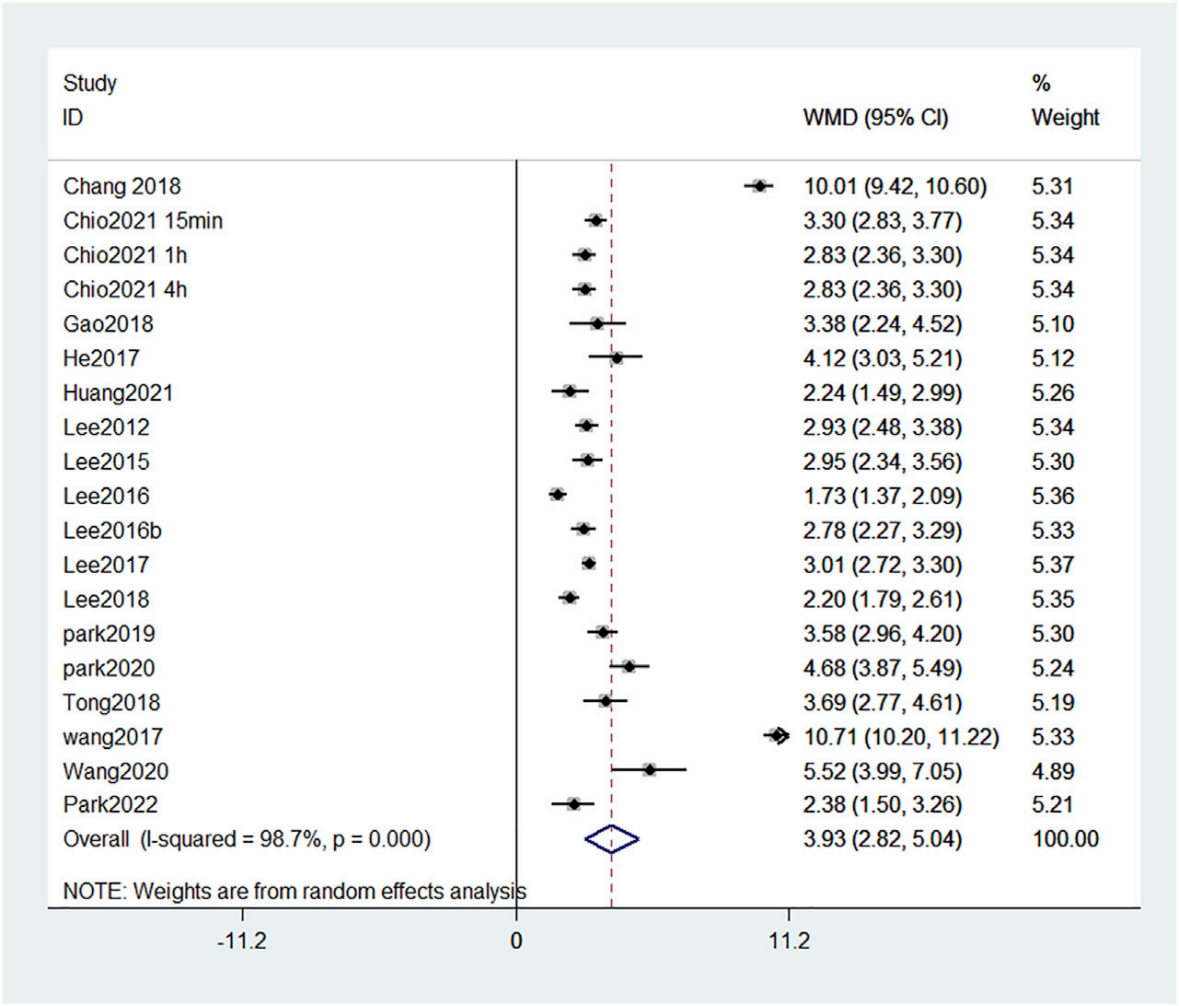
Forest plot of mean difference in BBB score after SCI and treatment at fourth week.

We identified 17 publications which measured the BBB score at the fourth week, and a forest plot showed that the total BBB score of the treatment group was 3.93 points higher than that of the SCI group at the same time point (Figure 5). This finding suggests that reducing the damage of BSCB can significantly improve motor function. Among them, the BBB score in Wang et al. (2017) and Chang et al. (2018) increased by 10 points. This indicates that the two treatments, GFP-HO-1CΔ23 and aFGF-HP hydrogels, are very effective at promoting functional recovery.

## 4 Discussion

We systematically review experimental therapies that have focused on mitigating BSCB injury after t-SCI over the past 10 years. Following strict inclusion and exclusion criteria, 28 publications were included, and the proportion of EB leakage attenuation of the main results and related secondary results were extracted for analysis. The effect of each treatment was accurately estimated by meta-analysis. After comparative analysis, we found that BMSC-Exos may be the most effective treatment for reducing BSCB damage by inhibiting the expression and activity of MMPs. At the same time, three related mechanisms (i.e., inhibiting the expression and activity of MMPs, inhibiting ER Stress, and regulating the Akt pathway) are crucial for protecting the BSCB.

Disruption of the BSCB has been suggested as a potential mechanism for the exacerbation of t-SCI. As a natural barrier between the spinal cord parenchyma and blood, the BSCB is a central nervous system barrier that controls and regulates the entry of foreign substances into the nervous system and maintains the homeostasis of that microenvironment (Albayar et al., 2019). The destruction of BSCB leads to the weakening of barrier function, and peripheral inflammatory cells such as macrophages and neutrophils can infiltrate the central nervous system through the damaged BSCB (Mun-Bryce and Rosenberg, 1998). Moreover, infiltration of inflammatory cells and increased expression of MMPs exacerbate the destruction of BSCB (Zhang et al., 2011). Although the exact mechanism associated with BSCB disruption is currently unknown, it is closely related to MMPs, ER stress, and the Akt pathway (Zhou et al., 2016a; Chang et al., 2018; Chang and Cao, 2021; Xin et al., 2021). Regulation of related proteins can significantly reduce EB leakage and increase TJ protein expression (Xin et al., 2021). Spinal cord EB leakage mostly occurred 1 day after t-SCI, indicating that BSCB damage is the most serious 1 day after t-SCI. In addition, the maximal neutrophil infiltration occurred 24 h after SCI (Donnelly and Popovich, 2008). Therefore, early treatment with drugs that inhibit BSCB destruction may help improve outcomes after t-SCI.

BMSC-Exos is a relatively easy-to-obtain bone marrow mesenchymal cell exosome. BMSC-Exos have a variety of neuroprotection-related cytokines such as transforming growth factor beta (TGF-β) and insulin-like growth factor 1 (IGF-1), which can improve the local environment of spinal cord injury (Luo et al., 2021). Previous studies have demonstrated its protective role in nervous system damage (Liu et al., 2021; Zhou et al., 2022). BMSC-Exos can significantly reduce the expression of MMPs, thereby protecting cell-to-cell junctions and further attenuating BSCB disruption *via* TIMP2. Although knockdown of TIMP2 could not completely eliminate the therapeutic effect of BMSC-Exos (Xin et al., 2021). BMSC-Exos treatment reduced EB leakage by 90%, while it increased ZO-1 and β-catenin the most. This shows that BMSC-Exos treatment can significantly reduce the damage of various factors to the intercellular connection after SCI by inhibiting the expression and activity of MMPs, thereby reducing the damage of BSCB.

MMPs belong to the zinc endopeptidase family, which degrade a variety of extracellular proteins such as extracellular matrix and play a role in matrix remodeling and wound healing. However, overactivation of MMPs is detrimental and it disrupts BSCB leading to blood cell leakage, apoptosis, and neurological dysfunction. Inhibiting MMP activation can alleviate the loss of Claudin-5 and degradation of the basement membrane, thereby effectively reducing BSCB damage (Lee et al., 2012). Although each study looked at different treatments, 39% of studies reported modulation of MMPs as an important factor. We found that inhibition of MMPs alleviates EB leakage by 39–90%. We found that the most effective treatment is subcutaneous injection of BMSC-Exos, which inhibit MMPs. BMSC-Exos mitigated EB leakage the most by 90% (Xin et al., 2021). Protocatechuic acid can reduce EB leakage by 78% by inhibiting the expression and activation of MMP-9 (Park et al., 2019). In addition, other related MMPs inhibitors such as MMP-8I, VPA, Ghrelin, WIB-801C, PCA, and GA can significantly reduce EB leakage and increase expression of AJ and TJ proteins. Inhibiting the expression and activity of MMPs can alleviate the degradation of Claudin-5 and basement membrane by MMP proteins after SCI, thereby exerting a protective effect on BSCB (Wei et al., 2022). In conclusion, inhibiting the activation of MMPs is a well-established and widely used approach to mitigate BSCB disruption. In the future, drugs that inhibit the action of MMPs in other systems, such as tumor and angiogenesis, can also be tried to reduce BSCB damage, which may provide new treatment strategies for BSCB damage.

In this review, although ER stress is not currently the most effective method, 29% of the publications included in this work mentioned that the disruption of BSCB is associated with ER stress. Recent studies have shown that ER stress inhibition prevents the degradation of AJ and TJ proteins (Mcguckin et al., 2011), attenuating secondary injury and promoting functional recovery after SCI (Lee et al., 2014b). Further, aFGF-HP attenuates BSCB disruption by preventing loss of TJs after SCI and reduced EB leakage by 66%, which is associated with inhibition of ER stress (Wang et al., 2017). Dl-3-n-butylphthalide (NBP) reduces the loss of TJ proteins in BSCB by inhibiting ER stress-related protein expression Therefore, modulation of ER stress-related pathways may provide a potential target for BSCB disruption in SCI therapy. The PI3K/Akt pathway is a common pathway regulating endothelial barrier permeability. Activation of the PI3K/Akt pathway attenuates ER stress (Hsu et al., 2017). Huang et al. (2021) demonstrated that activation of the Akt pathway can alleviate ER stress, thereby alleviating BSCB disruption caused by ER stress (Huang et al., 2021). It is possible that the protective effect of Akt pathway on BSCB is through regulating ER stress.

Significant recovery of neurological function was mentioned in most of the publications in this systematic review, among which, 17 reported that the BBB score increased by an average of 3.93 points 4 weeks after t-SCI. Reducing the damage of BSCB can reduce the infiltration of various harmful substances such as inflammatory cells, improve the local microenvironment, reduce the damage of neurons and axons, and provide a good environment for the regeneration of damaged axons (Wang et al., 2017; Gao et al., 2018). Interestingly, we found that the relative increase in BBB score was not significant for some treatments with strong BSCB protection. Carvacrol treatment reduced EB leakage by 80%, while the BBB score increased by only 2.38 (Park et al., 2022). In contrast aFGF-HP hydrogels treatment reduced EB leakage by only 66%, while increasing the BBB score most significantly, reaching 10.71 points (Wang et al., 2017). Therefore the use of hydrogel *in situ* administration can make the drug more concentrated on the injured site and provide a scaffold for axonal growth, which is more effective in promoting the recovery of neurological function in the later stage.

Different mechanisms of action can co-regulate the state of BSCB, and in future studies, we may combine the different mechanisms to achieve the best treatment strategies to alleviate BSCB damage. In addition, some new experimental techniques, such as the application of hydrogels, nanomaterials, and exosomes may generate more significant therapeutic effects. We can expect improved prospects for the treatment of BSCB disruption as new technologies continue to be applied.

## 5 Limitations

One of the limitations of this study, despite the selection of the time point for large BSCB leakage, was that we chose the first day after injury as the inclusion criterion and did not know the leakage at other time points after following SCI. However, at 24 h, BSCB leakage was the most obvious and was the most typical representative of BSCB damage. Another limitation is that although most studies have chosen the BBB score to assess motor recovery, the timing of their measurement of the BBB score varies. This makes it more difficult to compare the mitigation of BSCB damage and its contribution to improved motor function. Most animal studies of SCI have been performed in the T8-T10 segment, but human SCI occurs more often in the cervical spine. There are anatomical differences in the cervicothoracic segment, which will affect the clinical performance and functional recovery after injury damage, making clinical translation of the results more difficult. Another important limitation is the medium to high risk of bias in many studies, mostly due to the lack of clear reporting of relevant items. This can be addressed by referring to SYRCLE’s risk of bias tool in the reporting of animal experiments. In addition, in most publications data are presented as graphs, and our measurement process using software may be biased. In addition, when we use the error propagation formula to calculate the percentage of EB leakage mitigation, the error may be amplified by the formula.

## 6 Conclusion

Our results suggest that BMSC-Exos is currently the most effective treatment for alleviating BSCB injury. In addition, the regulation of MMPs, the Akt pathway and ER stress pathway can improve the damage of BSCB in t-SCI and promote recovery of neurological function.

## Data Availability

The original contributions presented in the study are included in the article/Supplementary Material, further inquiries can be directed to the corresponding author.
